# Supplementary material for: Fabrication of Humidity-Resistant Optical Fiber Sensor for Ammonia Sensing Using Diazo Resin-Photocrosslinked Films with a Porphyrin-Polystyrene Binary Mixture
Source: Sensors (Basel). 2021 Sep 15;21(18):6176. doi: 10.3390/s21186176 (PMC8469947; doi:10.3390/s21186176)
Supplement: Supplementary file 1 [file sensors-21-06176-s001.zip › sensors-1249395-supplementary.pdf]

Supporting information

Fabrication of Humidity-Resistant Optical Fiber Sensors for Ammonia Sensing Using Diazo Resin-Photocrosslinked Films with a Porphyrin-Polystyrene Binary Mixture

Soad Ahmed [a], Yeawon Park [a], Hirofumi Okuda [a], Shoichiro Ono [a], Sergiy Korposh [b], and Seung-Woo Lee [a,*]

[a] *Graduate School of Environmental Engineering, The University of Kitakyushu, 1-1 Hibikino, Kitakyushu 808-0135, Japan.*

[b] *Department of Electrical and Electronic Engineering, University of Nottingham, Nottingham, NG7 2RD, UK.*

*Correspondence: leesw@kitakyu-u.ac.jp (S.-W. L.); Tel.: +81-93-695-3293.

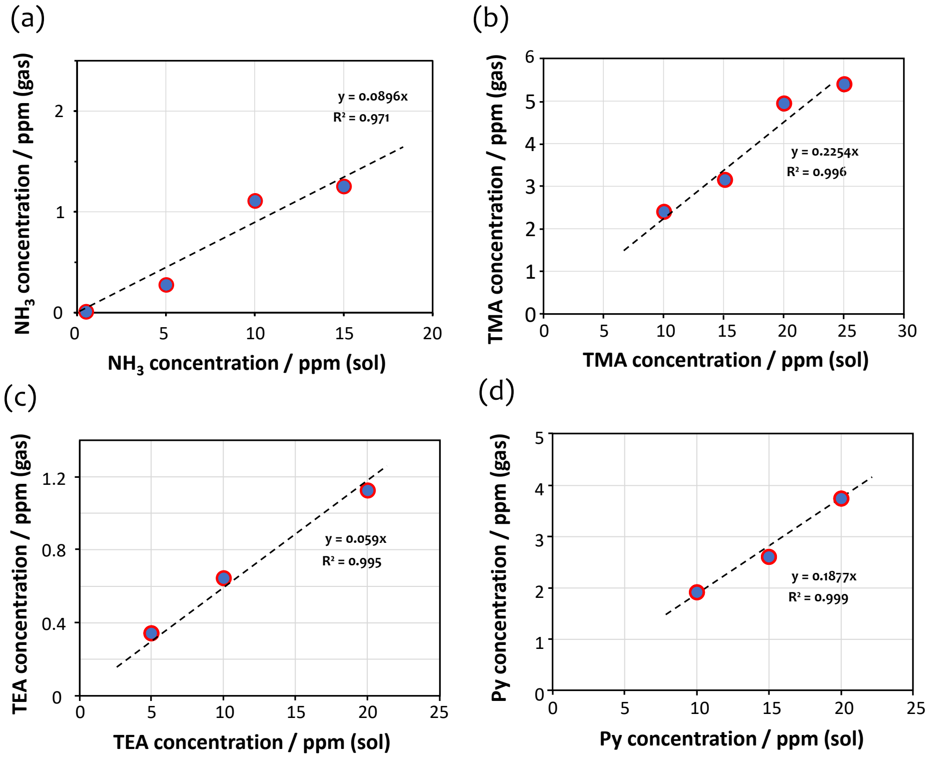

Figure S1. Calibration curves between the actual amine gas concentrations and the corresponding amine concentrations in the solutions: (a) ammonia, (b) TMA, (c) TEA, and (d) Py.

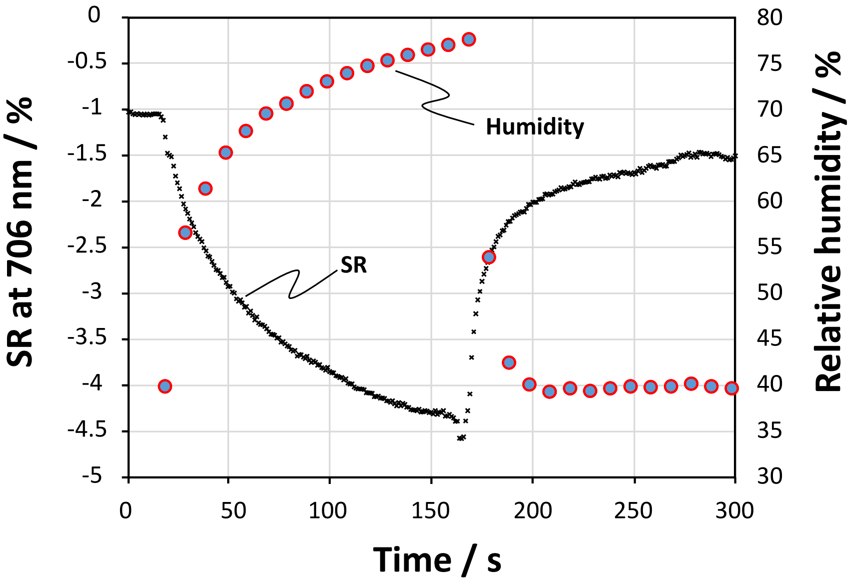

Figure S2. Relative humidity changes inside the measurement chamber during the sample measurement. When the SR, e.g., to 10 ppm (sol) of ammonia, almost saturated, the relative humidity reached approximately 80% at room temperature (approximately 24 °C).

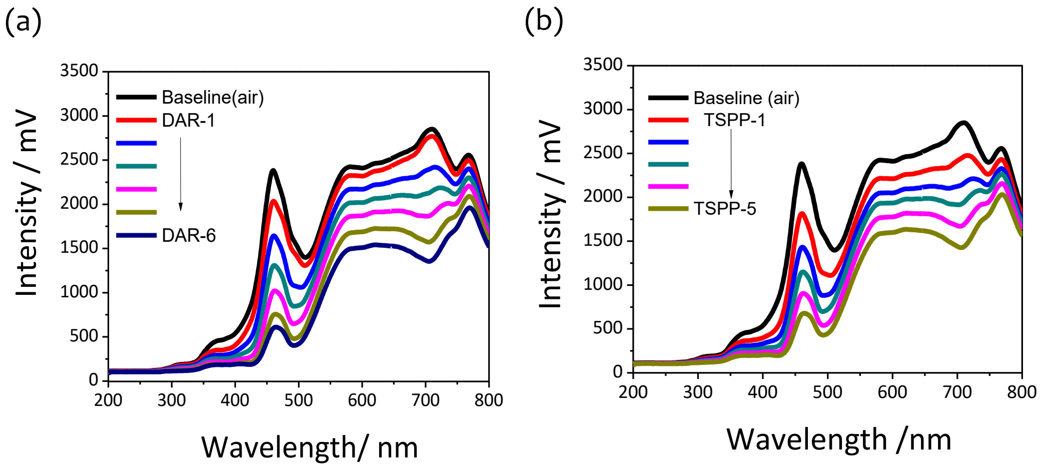

Figure S3. Evolution of the transmission spectra of the DAR/TSPP+PSS (0.025 wt%) alternate layers deposited onto the 1-cm-long stripped core of a U-bent optical fiber when the outermost layer was deposited with (a) DAR and (b) TSPP+PSS, respectively.

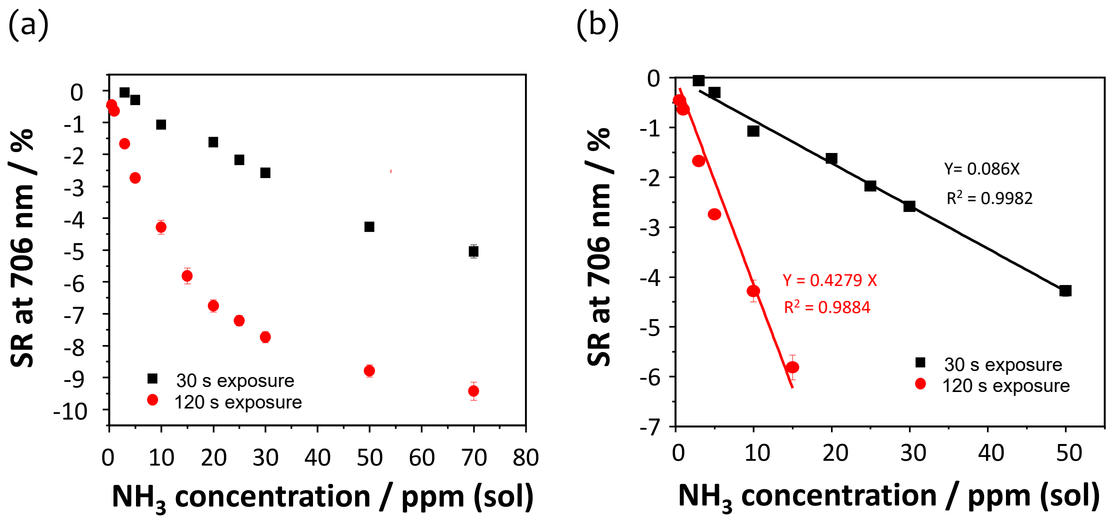

Figure S4. Comparison of the (a) SRs and (b) calibration curves of the OFS coated with a DAR/TSPP+PSS (0.025%) film when exposed to ammonia gas for 30 s and 120 s. The linear trend of the sensor response–concentration curve was extended up to 50 ppm (sol) of ammonia when the exposure time (30 s) was shortened.

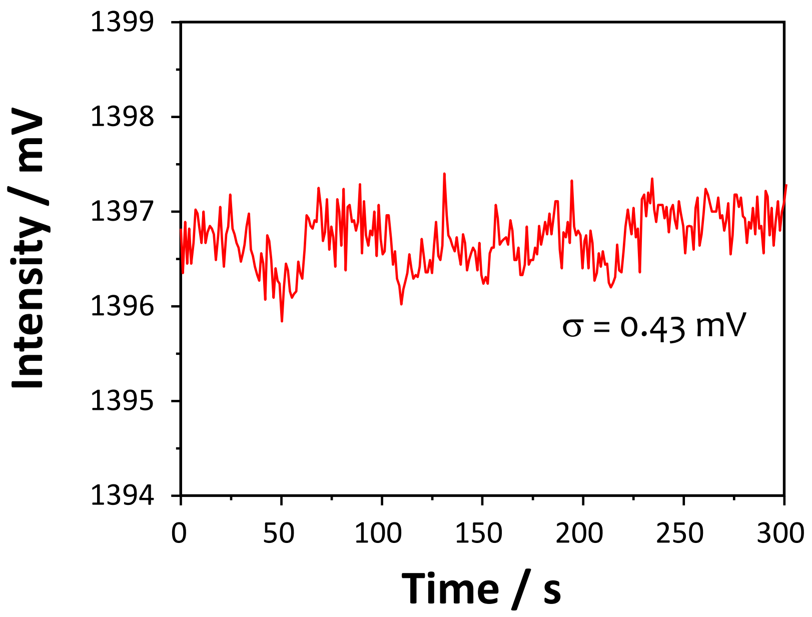

Figure S5. A baseline of the DAR/TSPP+PSS (0.025 wt%) film in a steady state before exposure to ammonia, showing a possible noise value of 0.43 mV (σ), which was used to determine the limit of detection (LOD) of the U-bent fiber sensors for ammonia.

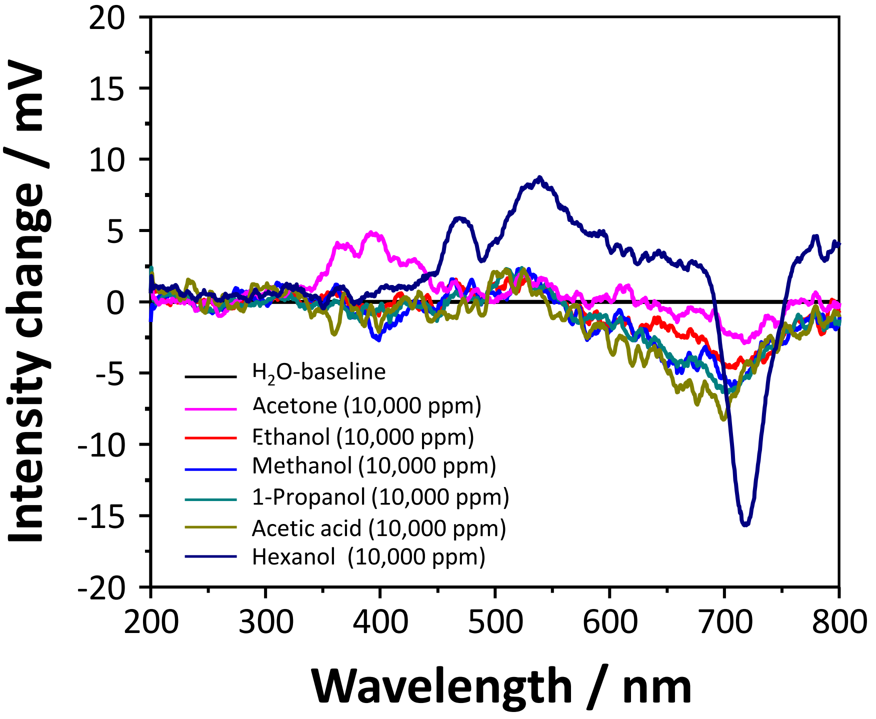

Figure S6. Intensity changes in the transmission spectra due to the exposure of the U-bent OFS coated with a DAR/TSPP+PSS (0.025%) to different non-amine analytes.

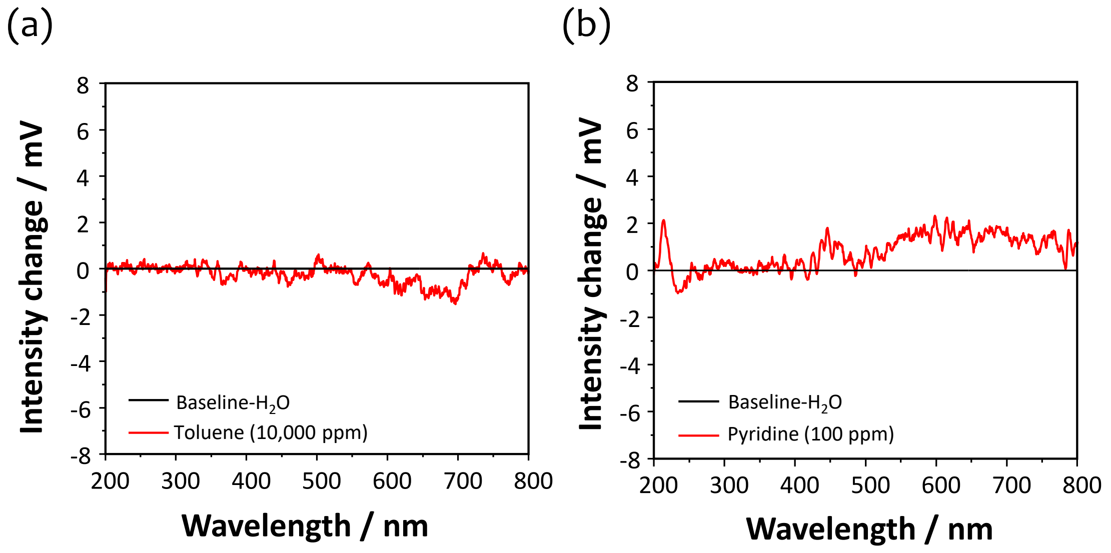

Figure S7. Intensity changes in the transmission spectra due to the exposure of the U-bent OFS coated with a DAR/TSPP+PSS (0.025%) to (a) toluene (10,000 ppm) and (b) Py (100 ppm).

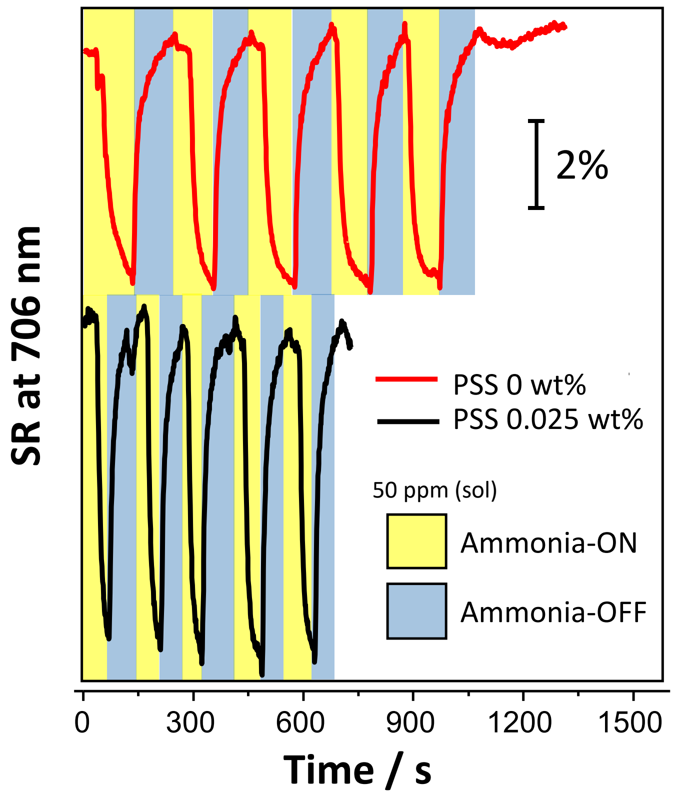

Figure S8. Comparison of dynamic SRs at 706 nm upon repeated exposure to 50 ppm (sol) of ammonia for the 5-cycle DAR/TSPP+PSS (0 and 0.025 wt%) films.

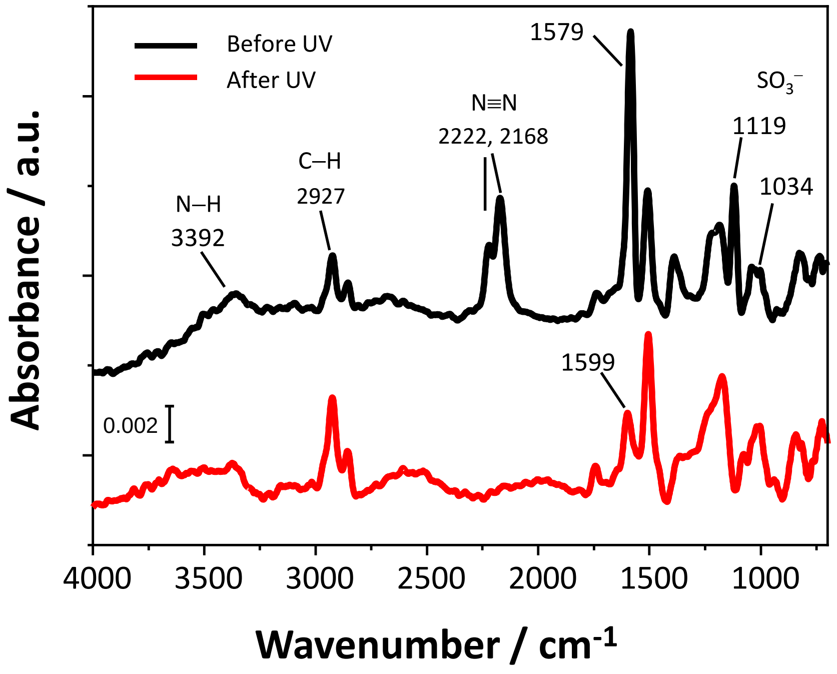

Figure S9. FT-IR spectra of a 10-cycle DAR/TSPP+PSS (0.025 wt%) film deposited on a gold-coated silicon wafer substrate before and after UV irradiation.

The formation of covalent linkages following the decomposition of the diazonium group was further verified by FT-IR measurements (Figure S9). A 10-cycle DAR/TSPP+PSS (0.025 wt%) film deposited on a gold-coated silicon wafer substrate was monitored before and after UV irradiation. The two absorption peaks observed before UV irradiation at 2168 and 2222 cm^{-1} originate from the symmetric and asymmetric stretching vibrational modes of the diazonium ion (N_2^+), respectively [1]. After UV irradiation, these two peaks disappeared completely, indicating the decomposition of the diazonium groups. The bands between 1500 and 1579 cm^{-1} are attributed to the aromatic rings among, and the strong peak at 1579 cm^{-1} indicates the presence of phenyl groups in the diazonium moiety of DAR. This characteristic peak decreased notably, with a subsequent peak shift to 1599 cm^{-1} after UV irradiation. The characteristic bands of sulfonate groups are usually observed in the range of wavenumbers from 800–1350 cm^{-1}. Thus, the two absorption peaks at 1034 and 1119 cm^{-1} can be attributed to the stretching vibrations of the aromatic sulfonate groups (SO_3^-). On the other hand, the peak at 1119 cm^{-1} completely disappeared, while the asymmetric stretching vibration of the sulfonate group at 1034 cm^{-1} became more apparent after UV irradiation. These results suggest that the successful decomposition of the diazonium group was achieved through the formation of a covalent bond between the DAR and the mixture of TSSP and PSS.

[1] Sun, J.; Wu, T.; Liu, F.; Wang, Z.; Zhang, X.; Shen, J. Covalently attached multilayer assemblies by sequential adsorption of polycationic diazo-resins and polyanionic poly(acrylic acid). *Langmuir* **2000**, *16*, 4620–4624, doi.org/10.1021/la991482z.
